# Recent Progress in Wearable Biosensors: From Healthcare Monitoring to Sports Analytics

**DOI:** 10.3390/bios10120205

**Published:** 2020-12-15

**Authors:** Shun Ye, Shilun Feng, Liang Huang, Shengtai Bian

**Affiliations:** 1 Microfluidics Research & Innovation Laboratory, School of Sport Science, Beijing Sport University, Beijing 100084, China; sjy5271@psu.edu; 2 Biomedical Engineering Department, College of Engineering, Pennsylvania State University, University Park, PA 16802, USA; 3 State Key Laboratory of Microbial Resources, Institute of Microbiology, Chinese Academy of Sciences, Beijing 100101, China; 4 State Key Laboratory of Transducer Technology, Shanghai Institute of Microsystem and Information Technology, Chinese Academy of Sciences, Shanghai 200050, China; shilun.feng@ntu.edu.sg; 5 School of Electrical and Electronic Engineering, Nanyang Technological University, Singapore 639798, Singapore; 6 School of Instrument Science and Opto–Electronics Engineering, Hefei University of Technology, Hefei 230009, China; lianghuang@hfut.edu.cn

**Keywords:** wearable biosensors, biomedical microfluidics, healthcare monitoring, sports analytics

## Abstract

Recent advances in lab-on-a-chip technology establish solid foundations for wearable biosensors. These newly emerging wearable biosensors are capable of non-invasive, continuous monitoring by miniaturization of electronics and integration with microfluidics. The advent of flexible electronics, biochemical sensors, soft microfluidics, and pain-free microneedles have created new generations of wearable biosensors that explore brand-new avenues to interface with the human epidermis for monitoring physiological status. However, these devices are relatively underexplored for sports monitoring and analytics, which may be largely facilitated by the recent emergence of wearable biosensors characterized by real-time, non-invasive, and non-irritating sensing capacities. Here, we present a systematic review of wearable biosensing technologies with a focus on materials and fabrication strategies, sampling modalities, sensing modalities, as well as key analytes and wearable biosensing platforms for healthcare and sports monitoring with an emphasis on sweat and interstitial fluid biosensing. This review concludes with a summary of unresolved challenges and opportunities for future researchers interested in these technologies. With an in-depth understanding of the state-of-the-art wearable biosensing technologies, wearable biosensors for sports analytics would have a significant impact on the rapidly growing field—microfluidics for biosensing.

## 1. Introduction

Miniaturization of laboratory apparatus into microscale devices is a promising technology called lab-on-a-chip (LOC) [1]. About 30 years ago the concept of micro total analysis systems (μTAS) emerged from the field of semiconductor fabrication and was enhanced by microelectromechanical systems (MEMS) technologies [2,3,4]. The μTAS concept is to shrink an entire analytical procedure, such as cell sorting, single-cell capture, captured-cell transport, cell lysis, and intracellular analysis, into a miniaturized multifunctional chip [5,6,7], and nowadays its well-known synonym is called lab-on-a-chip (LOC) [3,4]. This growing field has garnered considerable attention since scaled-down biochemical analysis has several key advantages over both conventional and current laboratory benchtop methods [3,5]. These advantages are consistently demonstrated in clinical medicine, engineering, biology, and life science, etc., for example, to expedite the experimental process by embracing automation and parallelization [1,8,9]; to lower the cost by reducing the volume of expensive reagents [1,5,10,11]; to yield better interpretation of experimental results by gleaning vital information at cellular even molecular levels [12,13,14]. 

Interest in device miniaturization [15,16,17], combined with advances in bio-microfabrication and enabling materials [18], is motivating various microfluidic methods in which microchips can be mass-manufactured at extremely low cost via polymers (e.g., polydimethylsiloxane, PDMS) and soft lithography for microfabrication [5,19]. Microfluidics is the science of microscale devices that process and manipulate extremely low (10^−9^ to 10^−18^ L) amounts of fluids in microchannels with dimensions of tens of micrometers [10]. Conventional macroscale experimental technologies meet difficulties to deal with such low amounts of fluids, impeding their development in various fields. Conversely, microfluidic technologies begin to address numerous tough challenges, because fluid phenomena at the microscale are dramatically different from those at the macroscale [3]. For instance, capillary forces and surface tension are more dominant than gravitational forces [3], allowing for passively pumping fluids in opposition to gravity [20]. Flows at the microscale are laminar instead of turbulent, resulting in more predictable liquid handling and diffusion kinetics [5]. Based on the different phenomena behaving at the microscale, microfluidic technologies offer a sensitive, predictable, and controllable avenue for bioanalysis [21].

Despite all the attractive capacities of LOC/microfluidics devices that have enabled the widespread implementation of microchip-based systems in biology and life science [3,4,5,22], microfluidic technologies often only improve the performance of existing macroscale assays or provide equivalent alternatives [13]. Conversely, they have not reached their full potential due to the lack of essentially new capacities [3]. In recent years, however, LOC/microfluidics technologies begin to address some problems that have not yet been solved by current laboratory benchtop methods. An excellent example can be found in wearable/ambulatory healthcare monitoring and sports analytics harnessing skin-interfaced wearable biosensors [15,23]. Although this field is still in its infancy, the fundamentals of it are exceptionally strong: in the past decade, the wearable LOC devices gradually integrated with well-established techniques, including biocompatible materials [24,25], flexible electronics [26,27,28,29,30], optical/electrochemical sensors [14,26,31,32], microfluidics [21,33,34,35], near-field communications (NFC) [36], pain-free microneedles [37,38,39,40], as well as big data and cloud computing [14,41,42]. 

**Figure 1:**
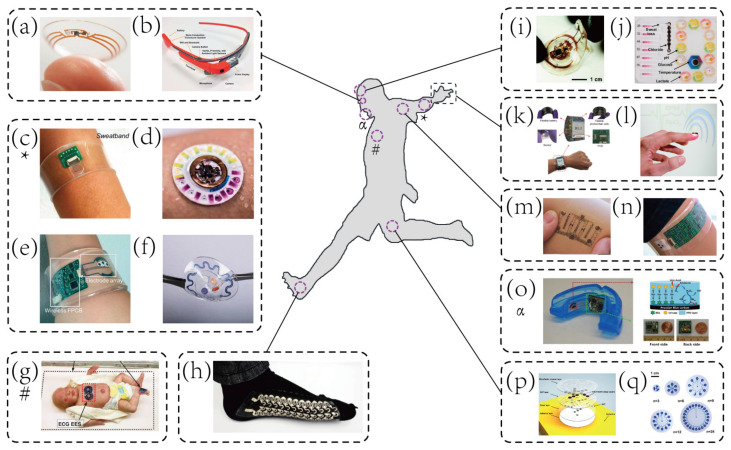
Representative examples of wearable biosensors for both healthcare and sports monitoring. (**a**) Contact lens sensors in ocular diagnostics [46]. Copyright 2015, Wiley. (**b**) Google glass for immunochromatographic diagnostic test analysis [87]. Copyright 2014, American Chemical Society. (**c**) A wearable microsensor array for multiplexed heavy metal monitoring [31]. Copyright 2016, American Chemical Society. (**d**) A hybrid sensor for simultaneous electrochemical, colorimetric, and volumetric analysis of sweat [73]. Copyright 2019, American Association for Advancement of Science. (**e**) A wearable sensor for autonomous sweat extraction and analysis [69]. Copyright 2017, National Academy of Sciences of United States of America (NAS). (**f**) A microfluidic device for colorimetric sensing of sweat [43]. Copyright 2018, American Association for Advancement of Science. (**g**) Binodal, wireless epidermal electronic systems with in-sensor analytics for neonatal intensive care [88]. Copyright 2019, American Association for Advancement of Science. (**h**) Wearable textile-based self-powered sensors [89]. Copyright 2016, Royal Society of Chemistry. (**i**) A microfluidic system for real-time tracking of sweat loss and electrolyte composition [27]. Copyright 2018, Wiley. (**j**) A microfluidic system for colorimetric analysis of sweat biomarkers and temperature [74]. Copyright 2019, American Chemical Society. (**k**) A smartwatch for continuous sweat glucose monitoring [72]. Copyright 2019, American Chemical Society. (**l**) A miniaturized battery-free wireless sensor for wearable pulse oximetry [26]. Copyright 2017, Wiley. (**m**) An epidermal stimulation and sensing platform for sensorimotor prosthetic control, management of lower back exertion, and electrical muscle activation [90]. Copyright 2016, Wiley. (**n**) A wearable electrochemical sensor for noninvasive simultaneous monitoring of Ca^2+^ and pH [32]. Copyright 2016, American Chemical Society. (**o**) A wearable salivary uric acid mouthguard sensor [58]. Copyright 2015, Elsevier. (**p**) A microfluidic system for time-sequenced discrete sampling and chloride analysis [91]. Copyright 2018, Wiley. (**q**) Skin-mounted microfluidic networks for chrono-sampling of sweat [63]. Copyright 2017, Wiley.

These above-mentioned enabling techniques establish the foundations for a new generation of wearable biosensors that directly interfaced with the human epidermis instead of rigid packages embedded in wrist straps or bands [23,43,44,45]. The distinguishing characteristics of the emerging wearable biosensors, lightweight, flexibility, and portability [31,36,46], have made them especially suitable for point-of-care testing (POCT). Therefore, brand-new wearable biosensors capable of real-time physiological monitoring quickly emerge, as shown in Figure 1. However, these wearable biosensors are mainly designed for health monitoring [15,34,41,45,47,48], especially, some of them are only developed to measure the physical strain/stress bending change [25,49,50]. Although many wearable devices have been deployed in sports, they are used to monitoring biophysical markers [23], such as movement [51] and cardiovascular information (e.g., blood oxygenation) [26,52,53]. 

Conversely, wearable biosensors are relatively underutilized resources to gain biochemical insights about athletes’ health status. The POCT microfluidic devices have been used to analyze biofluids, such as urine [31,32,54], tears [46,55,56], saliva [57,58,59,60], and sweat [27,43,61,62]. However, for the applicability of wearable biosensing, not all biofluids show on-field potential. 

Biofluids for physiological/pathological information acquisition are listed as follows. Their suitability for wearable biosensing is also discussed.

1. Sweat: Sweat is a biofluid produced and excreted by the eccrine glands within the epidermis, containing numerous important biomarkers that could be detected and assessed through the non-invasive collection and biochemical analysis [27,43,45,61,62]. For example, wearable microfluidic/electrochemical sweat biosensors are able to detect various electrolytes, such as sodium ions (Na^+^) [14,61,63,64,65,66,67], potassium ions (K^+^) [14,63], calcium ions (Ca^2+^) [32], and ammonium (NH_4_^+^) [68], multiple metabolites, such as glucose [14,37,69,70,71,72], lactate [14,21,63,73,74,75], several heavy metal species, such as zinc, iron, copper, and magnesium [61,76], and drug contents, such as Levodopa [77] (more sweat analytes can be found in Section 5.1 and Table 1). As such, sweat contains a wealth of physiologically relevant information [41,43,45] that can reflect hydration [21,27,33,74], electrolyte balance [63], exercise intensity [78], renal function [34,79], etc. For diagnostic use, wearable sweat biosensors are used to diagnose cystic fibrosis (CF), liver diseases, kidney disorders, as well as to monitor stress levels by measuring the cortisol concentrations in sweat [34,43,80]. Nonetheless, wearable biosensors capable of real-time biochemical analysis are still at an early stage of development. For instance, challenges still exist in extracting and calibrating the concentration of biomarkers in sweat due to regional variations and individual hydration status. Besides, other daunting challenges will be discussed in Section 6. In this paper, an in-depth overview of wearable sweat biosensing will be presented since sweat is the most well-studied analyte source among all six typical biofluids in the wearable biosensing field.
2. Interstitial fluid (ISF): ISF is an attractive biofluid presented in the human dermis, and has rich analytes from blood, especially through capillaries. Due to the ease of fluid exchange, lots of analytes have near levels of concentration between ISF and blood [81]. The microneedle patches have many diagnostic applications via ISF manipulation [82]. The MNs are the miniaturized replica of hypodermic needles aimed at minimally-invasive transdermal ISF biosensing [37,83] without blood sampling, which will be briefly reviewed in Section 5.3.1.
3. Saliva: Saliva has been recognized as an alternative to blood, too [57,58,59,60]. Non-invasive analysis of fluoride (F^-^), Na^+^, pH, and uric acid has been demonstrated [84]. The mouthguard platforms combined with electrochemical biosensors for monitoring were developed for wearable salivary monitoring of metabolites [85]. However, salivary monitoring may be affected by huge amounts of microbes (e.g., bacteria) in the oral cavity that could cause specimen contamination [86].
4. Tears: Recently, the contact lens has attracted considerable interest as a platform for in situ biosensing of tear fluid [46,56]. Research shows that glucose, Na^+^, and K^+^ can be found in tears and tear fluid is less complicated than blood due to the presence of the blood-tear barrier [55]. Unfortunately, tear fluid has a relatively low potential for wearable biosensing because of the low diversity of analytes and a strong reliance on proximal wireless power delivery.
5. Urine: Urinalysis has been widely used as a means to monitor the overall health status and screen various diseases due to ease of non-invasive collection and relatively large amounts [31,32,54,85]. Despite these advantages, urinalysis still has difficulties performing on-field wearable biosensing due to the difficulty of calibration of concentration levels of analytes levels, because they are strongly related to individuals’ hydration levels.
6. Blood: It is known that blood analysis is usually not suitable for wearable sensing due to its invasive nature, skin-piercing for blood sampling. Only several recent studies demonstrate its utility in wearable biosensing. These studies harnessed fingernail-mounting optoelectronic biosensors to in situ continuous monitoring of vital signs [26,52,53]. A representative example [26] will be discussed in Section 4.2.4.

Overall, although the underlying values of wearable biosensing still need to be proven and demonstrated, sweat and ISF biosensing show promise that may not be recognized by researchers. Skin offers a non-invasive (sweat) and minimally-invasive (ISF) diagnostic interface rich with biological insights from our inner body [21,47]. Thus, to demonstrate the potential value of wearable biosensors for health-related and sports analytics by reviewing recent progress is required. Besides, due to the space limitations, the wearable biosensors for interrogating ISF [37,83], saliva [58], and blood [26] are discussed in a limited manner. Wearable biosensing modalities for tracking non-sweat biofluids (tears and urine) are not included, they could be found in recent reviews [15,44,81].

In this review, the latest developments in wearable biosensors will be reviewed in detail, from materials and fabrication strategies, sampling modalities, sensing modalities to key analytes and wearable biosensing platforms for health and sports monitoring with an emphasis on sweat and ISF biosensing. Afterwards, the unsolved challenges for future researchers specializing in diverse fields (e.g., biomedical/electronic engineering, biochemistry, healthcare, pharmaceutics, sports science, etc.) will also be briefly discussed. A concluding section summarizes the trend towards wearable biosensing for sports analytics and forecasts promising breakthroughs in the rapidly growing field—microfluidics for biosensing.

## 2. Materials and Fabrication Strategies

### 2.1. Materials

Materials for Substrate: the softness and attachability of the substrate are the main concerns for the stretchability of wearable biosensors, and directly determine the comfort of the biosensor and the reliability of long-term monitoring. Common base materials are organic materials such as polymers, silicones, and rubbers. For example, polydimethylsiloxane (PDMS) has inherent high stretchability, non-toxic, hydrophobic, and good workability. It has been used in microfluidics, prostheses, and wearable biosensors [92]. Polyethylene terephthalate (PET) is characterized by lightweight and can serve as a flexible substrate for ink-jet printed electronics [93]. Polyimide (PI) film is another commonly used substrate. It can maintain flexibility, creep resistance, and tensile strength under high temperature (up to 360 °C) and acid/alkali conditions [94]. Cellulose paper is a flexible, porous, inexpensive, recyclable, biodegradable, and biocompatible substrate material, and it has been widely used in test strips for medical diagnosis [95,96].

For attachability, substrates for skin-mounted biosensors need to meet strict requirements, including the wearable power source, prolonged wear time, and facile and painless removal after use without leaving residues. Meanwhile, they should be safe and minimize skin irritation and damage, such as epidermal stripping, blisters, skin tears, irritant contact dermatitis, allergic dermatitis, maceration, and folliculitis [97,98]. Polymeric materials, including acrylics, hydrocolloids, hydrogels, polyurethanes, rubbers, and silicones, have been used as the adhesive for skin-contact applications. Adhesives can be categorized into structural adhesives and pressure-sensitive adhesives (PSA). Materials for structural adhesives include cyanoacrylates, urethanes, epoxies, and acrylics, which lend themselves to structural applications. PSAs should deform easily under small pressures and can adhere to wet surfaces [99]. Aggressive adhesives based on acrylics, hydrocolloids, hydrogels, natural/synthetic rubbers, and polyurethanes can cause skin trauma (such as skin stripping, maceration, and allergic reactions), as well as leave residue on the skin [100]. Besides, soft silicone-based adhesives have good biocompatibility, temperature stability, chemical inertness, and environmental stability. These features make silicone-based adhesives suitable for many medical applications, including tapes, wound dressings for wearable devices, and transdermal drug delivery applications [101,102]. 

Materials for active element: (a) Carbon materials have been widely used in wearable sensors, and mainly include graphite, carbon nanotube (CNT), and graphene. Graphite is often used to form pencil—paper electrode, drawing patterns on the substrate by pencil painting [103]. CNT is a carbon material with extraordinary conductivity and mechanical strength [104,105]. Although a single CNT has a high sensitivity to strain, it is constructed and used on a larger scale. Therefore, CNT powders are often mixed into polymer substrates to realize wearable sensing. Compared with graphite and CNT, graphene has better electrical conductivity and mechanical properties, and has been widely used in flexible/stretchable sensors [106]. (b) Metal materials with excellent electrical conductivity have been widely used in wearable biosensors. (1) nanowires (NWs) or nanoparticles (NPs); NWs and NPs are often used as fillers to prepare piezoresistive composites and conductive ink [107,108]. For instance, AgNWs can be embedded into PDMS to build resistive-type sensors, and conductive inks with metal NPs can be cast and annealed on the substrate surface to form the electrodes for capacitive sensors. (2) Flexible or stretchable configurations with stretch structures; the flexible or stretchable configuration utilizes coiled buckled, serpentine and woven structures to make metal flexible and stretchable [109,110]. (3) Liquid metal; liquid metal utilizes its liquid state at room temperature and combines with microfluidic technology to achieve electrode fabrication [111,112]. (c) Polymers have been widely explored in sensing elements for their thermal stability, high transparency, and tunable conductivity, such as poly 3,4-ethylene dioxythiophene (PEDOT) [113] and polyvinylidene difluoride (PVDF) polymers [114].

### 2.2. Fabrication Strategies

According to the materials that can be used for flexible and stretchable sensors, the fabrication methods are categorized into compositing materials and pattern transferring. (a) Mixing different materials (e.g., carbon black NP, AgNW) into the composite is the simplest manufacturing method [115,116]. The active material is blended into the polymer by stirring, and then can be made into a dry elastic composite material in bulk or film form. Mixed composites have complex electromechanical features, relying on fillers and polymer substrates and doping concentration and distribution state. (b) Pattern transferring is the most common manufacturing method to produce the desired pattern of wearable biosensor geometries. It mainly includes microscale modeling, lithography, printing, and handwriting. Microscale modeling is used to prepare the substrates, electrodes, and sensing microstructures in composite materials. Lithography is a pattern transfer method that can realize various novel geometric shapes in flexible electronic products. Lithography technology specializes in manufacturing complex stretchable systems with high-precision dimensions, exquisite structure, and rich functions [117,118]. Printing can deposit and pattern many materials on various substrates at the same time without the need for complicated equipment and clean rooms. Screen printing is a typical printing technique that required masks [119,120]. This technology has been widely adopted to fabricate working electrodes in electrochemical sensors and the sensing elements in electromechanical sensors. The handwriting method is a technique in which various instruments are used to directly draw electrodes on the substrates, which has become an alternative for manufacturing low-cost, DIY sensors [96,121]. This technology gives end-users the ability to design and implement sensors based on “on-site, real-time” requirements. With a more complex structure, rollerball pens and fountain pens can be written with a variety of inks (including metallic inks, liquid metals, and organic mixtures) to generate controllable geometric shapes on many substrates. In addition to screen printing technology, inkjet printing technology is also an important technology that has been widely used in sensor fabrication [122,123]. Inkjet printing is a precise and fast film preparation technology, which sprays functional ink droplets onto paper or other substrates through nozzles. The materials used include carbon nanotube, graphene, conductive metal solution, polymer, and liquid metal, and other functional inks [124,125].

## 3. Sampling Modalities

**Figure 2:**
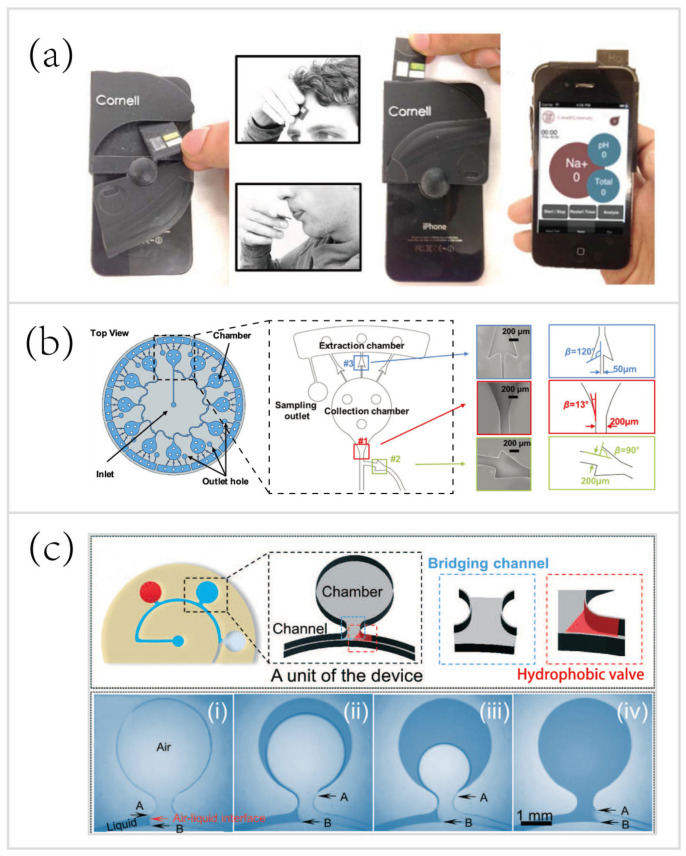
Representative examples of sweat sampling modalities. (**a**) A smartphone-based sweat biosensor, consisting of a disposable test strip for sweat sampling via direct contact [126]. Copyright 2013, Royal Society of Chemistry. (**b**) A microfluidic biosensor with capillary bursting valves (CBVs) for sampling sweat in a sequential manner and with an interconnected set of chambers for the temporary storage of sweat samples for subsequent ex-situ analysis of sweat biomarkers [63]. Copyright 2017, Wiley. (**c**) A skin-interfaced microfluidic biosensor with sweat collection chambers and hydrophobic valves (HVs) for chrono-sampling of sweat; (**i**)—(**iv**) A series of images during the fluid sampling for demonstrating the working principle of HVs [128]. Copyright 2020, Royal Society of Chemistry.

To lay foundations for various biosensing modalities, including optical and electrochemical sensing, sampling modalities will be introduced. The majority of sweat analytical methodologies mainly adopt three sampling methods, direct contact, chamber collection, and multistep sampling. For example, paper-based methods, such as disposable test strips, have the devices directly contact the human epidermis to sample biofluids in just a few seconds. As shown in Figure 2a, Oncescu et al. [126] developed a smartphone-based biosensing system, which consisted of a disposable test strip for sweat/saliva sampling, a back compartment for eliminating the interference from ambient lighting conditions to improve the accuracy of colorimetric sensing, and a smartphone application for measuring biomarkers by digital image analysis and displaying the results to end-users. 

Another sampling method concerns chamber collection. The microfluidic systems using this sampling method have microchannels for guiding the sweat to fill on-chip microchambers for biochemical/enzymatic reactions to yield measurable color/potential changes [34,61,74,127]. The chamber collection method for sweat sampling and subsequent in situ biomarker analysis has been widely adopted, thus examples and corresponding technical details will be thoroughly reviewed in the following sections (Section 4.2.1 and Section 4.2.3). 

The last sampling method, multistep sampling, also has its applications in microfluidic biosensing. In the first step, various microchambers are also exploited for in situ sweat collection. However, unlike most microfluidic systems conducting in situ analysis, these microchambers are specially designed for the temporary storage of sweat but not for on-chip analysis. Microfluidic valves, such as capillary bursting valves (CBVs) (Figure 2b) [63] and hydrophobic valves (HVs) (Figure 2c) [128], are exploited to carry out sweat collection and storage in a time-sequential manner by microfluidic networks with interconnected sets of microchambers. The CBVs and HVs open only until the microchambers set before them have filled with sweat, enabling passively guiding sweat through the microfluidic network sequentially. After sweat sample acquisition, there is another step to transport the sweat for ex-situ laboratory analysis. For instance, Choi et al. [63] developed a skin-interfaced microfluidic chip with CBVs for chrono-sampling and ex-situ biomarker analysis of sweat (Figure 2b). CBVs were exploited to collect sweat in a time-sequential manner. Afterwards, centrifugation and retrieval of collected sweat from chambers allowed for chemical analysis of sweat biomarkers (lactate, Na^+^, and K^+^) by mass spectrometry.

## 4. Sensing Modalities

Real-time tracking of the physiological status of patients or athletes by wearable biosensors is essential to provide insightful information about their health conditions, sports performance, cardiovascular capacities, and so on. As described previously, the progress in enabling materials and fabrication strategies further improves both mechanical features (flexible, stretchable, non-irritating) and performance (selectivity, sensitivity, durability, reproducibility, accuracy) of wearable sensors. Several microfluidic, electrochemical, and optical biosensors demonstrate greater functionality. The main two sensing modalities, including electrochemical and optical sensing, which make optimum use of these systems for wearable in situ analysis of biofluids (e.g., sweat, saliva, and blood), will be systematically reviewed in this section. Other sensing modalities will also be briefly discussed.

### 4.1. Electrochemical Sensing

Early developed wearable biosensors mainly focused on detecting a single parameter, such as sodium concentrations [64]. Recently, multianalyte tracking changes in biofluid chemistry becomes a widely available biosensing technique since the upsurge of the electrochemical sensing methods, including potentiometry, conductometry, and also voltammetry/amperometry [45].

#### 4.1.1. Potentiometry

**Figure 3:**
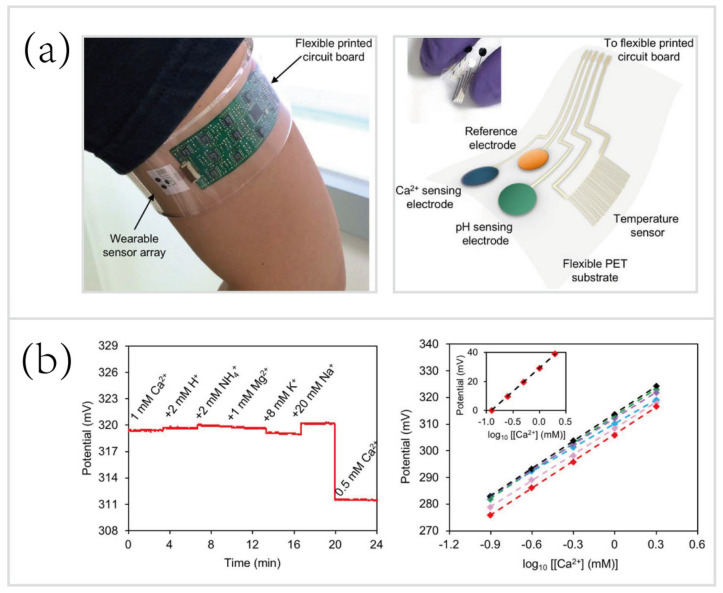
A fully integrated electrochemical biosensor for non-invasive in situ analysis of biofluids. (**a**) An image and a schematic illustration of a fully integrated potentiometric biosensor, consisting of Ca^2+^, pH, and temperature sensors patterned on PET substrate (**b**) Performance test results of Ca^2+^ sensors: ion-selectivity and reproducibility (n = 6) [32]. Copyright 2016, American Chemical Society.

Potentiometry is a highly sensitive, selective, reproducible electrochemical sensing method that has been exploited for wearable, non-invasive monitoring of biofluids (e.g., sweat, urine, and tears). The potentiometric biosensor usually contains several ion-selective electrodes (ISEs) and a reference electrode (RE) [14]. The potential of the RE is independent of the analyte concentrations, and the potential of ISE, conversely, depends on them. Eventually, the differences between ISEs and RE are used to determine the concentrations of targeted analytes. The research group at the University of California (Berkley) developed a wearable potentiometric biosensor for in situ monitoring of Ca^2+^ and pH [32]. As shown in Figure 3a, a PET-based flexible biosensor array of ionized calcium and pH was interfaced with a flexible printed circuit board (FPCB). Measurements of the concentrations of Ca^2+^ and pH were based on ISEs, coupled with an Ag/AgCl RE. An interfacing signal conditioning circuitry was used to measure the electrical potential differences between the ISEs and a RE, that were proportional to the logarithmic concentration of respective target ions. As mentioned previously, sweat contains a wealth of electrolytes, such as Na^+^, K^+^, and NH_4_^+^. Thus, the selectivity of potentiometric biosensors is of utmost importance. To demonstrate the high selectivity of this platform, H^+^, NH_4_^+^, Mg^2+^, K^+^, and Na^+^ were added to the Ca^2+^ solution sequentially. However, as shown in Figure 3b, the potential changes due to the addition of non-targeted ions were significantly smaller than due to the Ca^2+^. The performance test results (Figure 3b, right) also showed its high reproducibility (n = 6, RSD = 1.5%; RSD, relative standard deviation). Moreover, electrochemical sensing modalities also allow for seamless system integration, the FPCB of this biosensor integrated signal transduction, conditioning, processing, and wireless data transmission chipsets, eliminating delayed sample processing compared to traditional benchtop methods.

#### 4.1.2. Conductometry

Conductometry is another electrochemical sensing modality, but it has not yet been applied to wearable biosensing due to the reliance on high-capacity power sources and bulky electronics [45]. Few conductometric sensors were used to analyze biofluids in the literature. Ogasawara et al. [129] reported a flexible conductimetric sensor for ocular disease diagnosis by measuring the electrical conductivity of tears. There is a significant difference (*p* < 0.01) between electrolyte concentrations of healthy individuals and keratoconjunctivitis sicca (KCS) patients, which can be used to diagnose KCS patients without ocular damage by conductometric sensing. 

#### 4.1.3. Voltammetry/Amperometry

**Figure 4:**
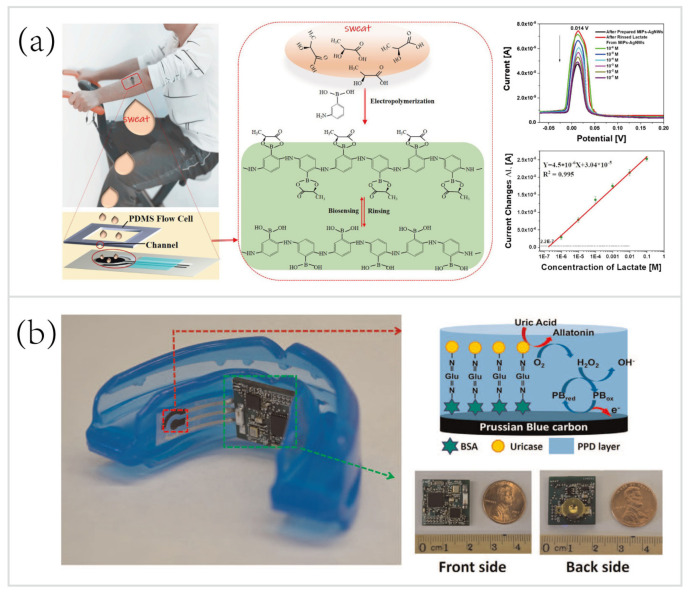
Wearable voltammetric/amperometric biosensors for sweat/saliva analysis. (**a**) A wearable voltammetric biosensor based on Ag nanowires (AgNWs) and molecularly imprinted polymers (MIPs) for in situ monitoring of lactate in the human sweat. A three-electrode MIPs-AgNWs biosensor mounting on the volunteer’s arm; the principle of MIPs’ formation and biosensing on the carbon working electrode; differential pulse voltammetry (DPV) responses and the calibration curve of MIPs-AgNWs biosensor for detection of lactate [127]. Copyright 2020, Elsevier. (**b**) A wearable amperometric mouthguard biosensor for real-time continuous salivary uric acid detection, screen printed uricase carbon working electrode, and its fabricated printed circuit board assembly (PCBA) [58]. Copyright 2015, Elsevier.

Voltammetry and amperometry are two rapid, real-time methods for detecting a milieu of analytes, such as lactate and heavy metals [31,76,127]. Voltammetric/amperometric biosensors have at least one three-electrode configuration, which includes a working electrode (WE), an RE, and a counter electrode (CE) [45]. Especially for voltammetric sensing, a time-dependent varying voltage with respect to the RE is applied, and the response between the WE and the RE is scanned within a range to oxidize/reduce the analytes. The peak heights or amplitudes of local maxima in the measured current are exploited to determine the concentrations of the analytes. For example, Zhang et al. [127] reported a wearable electrochemical biosensor based on molecularly imprinted Ag nanowires (AgNWs) for in situ monitoring sweat lactate (Figure 4a). The majority of sweat lactate electrochemical biosensors employ enzymatic reactions for gleaning detectable electrochemical signals. However, some environmental factors (e.g., temperature) can affect the activity of enzymes. 

Molecularly imprinted polymers (MIPs) were introduced for in situ detection of lactate in sweat, eliminating the need for proper storage of enzymes. This novel skin-mounted biosensor consisted of a three-electrode configuration: a MIPs-AgNWs coated carbon WE for providing specific binding sites to sweat lactate molecules, an Ag/AgCl RE, and a carbon CE. As shown in Figure 4, differential pulse voltammetry (DPV) responses, especially the peak current changes, were recorded for sensing of sweat lactate at concentrations from 10^−6^ M to 0.1 M. Considerable interest in sweat lactate stems from its positive correlation to the exercise intensity [78], which can be used to evaluate the athletic performance. Thus, this AgNWs-MIPs biosensor is highly applicable for sports analytics, especially during endurance, high intensity, or high strength activities. 

Similar to voltammetry for wearable sweat biosensing, amperometry has been used for electrochemical biosensing of saliva [58,84]. Mouthguard electrochemical sensors have been established for wearable biosensing of saliva analytes, such as glucose [59], lactate [84], uric acid [58], and bacteria [57]. For example, in 2015, Kim et al. [58] presented a wearable mouthguard biosensor for real-time monitoring saliva biomarker, uric acid, in a highly sensitive, selective manner (Figure 4b). The WE, RE, and CE were screen-printed on a flexible PET substrate and the Prussian-Blue carbon WE underwent chemical modification—crosslinking uricase and o-phenylenediamine (OPD). The PET-based sensor was integrated with an amperometric circuitry—miniaturized fabrication printed circuit board assembly—that consisted of a Bluetooth low energy communication System-on-Chip. The resulting wearable mouthguard biosensor showed high sensitivity to uric acid and had a wide linear range (covering both healthy and hyperuricemia people’s salivary uric acid concentration levels) (R^2^ = 0.998). Besides, saliva’s suitability of non-invasive monitoring has been proven in previous literature, a good correlation between blood and saliva concentration of uric acid have been found [130,131]. This wearable salivary biosensing platform can be rapidly expanded to multiparameter detection of saliva analytes and would have various real applications in the future.

### 4.2. Optical Sensing

#### 4.2.1. Fluorometry

**Figure 5:**
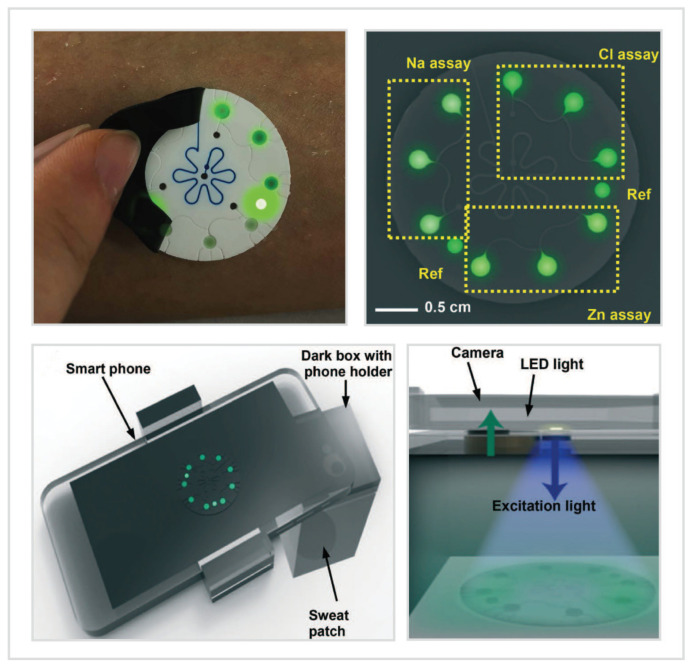
A fluorometric microfluidic biosensor for the detection of sweat biomarkers. Digital image of a fluorescent-based microfluidic device for in situ sensing the concentrations of sweat chloride, sodium, and zinc; fluorescent image illustrating the signals associated with targeted biomarkers; schematic illustrations of the smartphone-based optical module for fluorometric sensing [61]. Copyright 2018, Royal Society of Chemistry.

Fluorometric sensing is a simple approach to circumvent the need for in situ processing and data transmission, and also the on-chip power supply. The fluorescent-based biosensor only serves as the sweat sampler and in situ bioreactor that generates measurable changes in fluorescent intensity. Although fluorometry relies on visual readout devices (e.g., mobile phone), this sensing modality not only reduces the complexity of the wearable biosensor, it also lowers the cost of mass-manufacture of the device due to the simple structure without embedded electronics. Besides, the intrinsic merits of fluorescent-based biosensors are high sensitivity, selectivity, and accuracy. Fluorometric sensing exploits the chemical probes which interact with the target analytes and emit measurable fluorescence. The fluorescence is excited by a given light source (e.g., light-emitting diode, LED) and quantified by a photodetector (e.g., smartphone camera). Sekine et al. [61] presented a ground-breaking microfluidic biosensor using fluorometric sensing to determine the concentrations of sweat chloride, sodium, and zinc (Figure 5). Sweat was routed from inlets to fill the microchambers with fluorometric reagents time-sequentially under the control of CBVs. After the mixture of sweat and fluorescent probes, the light-shielding film over the fluorometric microchambers was removed and the smartphone-based optical module was exploited to capture the fluorescent images of the microchambers. The optical module consisted of a smartphone and a dark box, which contained a blue excitation filter to delineate the range of wavelengths of the excitation light and a colored emission filter to enable detection of excited fluorescence intensities without interference from the excitation light. Meanwhile, the two-round microchambers, consisting of a mixture of an ionic liquid of known concentration and the fluorometric reagents, served as fluorescence reference makers. Finally, the fluorescent intensities of fluorometric microchambers measured by ImageJ were used to determine the concentrations of sweat chloride, sodium, and zinc. The results of human trials in field tests strongly correlated with those measured by conventional benchtop methods. Hence, fluorometric sensing avoids the interference of ambient light conditions, leading to higher accuracy, but has a minor drawback—increasing the reliance on the optical module (e.g., dark box).

#### 4.2.2. Bioluminescence

Bioluminescent (BL) sensing has been widely used for the detection of pollutants and toxicants, including organic chemicals (e.g., benzene, toluene) [132] and heavy metals (e.g., lead, zinc, cadmium, copper, and mercury) [132,133,134], in water, food, or the environment, especially for environmental toxicology and bioterrorism controls. Compared to fluorometry, which requires external excitation light [135], BL exploits genetically engineered luminous cells/bacteria to detect specific compounds by measuring the BL signals produced by these recombinant cells/bacteria in the presence of the analytes [132,134,136,137]. BL bacteria emit light in a hospitable environment but with decreasing BL levels in the presence of the increasing amount of the toxicants. The weaker the light emitted by BL bacteria, the higher the degree of toxicity [132]. However, bioluminescence has not yet been used for tracking analytes in biofluids due to the reliance on living cells/bacteria. But, when the storage and maintenance of BL cells/bacteria as well as the sensitivity of BL sensors, are greatly improved, in the future, BF sensors may be used for rapid in situ analysis of biomarkers by integrating wearable technology. Here, a portable BL biosensor will be discussed to further illustrate the principles and show the potential of bioluminescent sensing.

**Figure 6:**
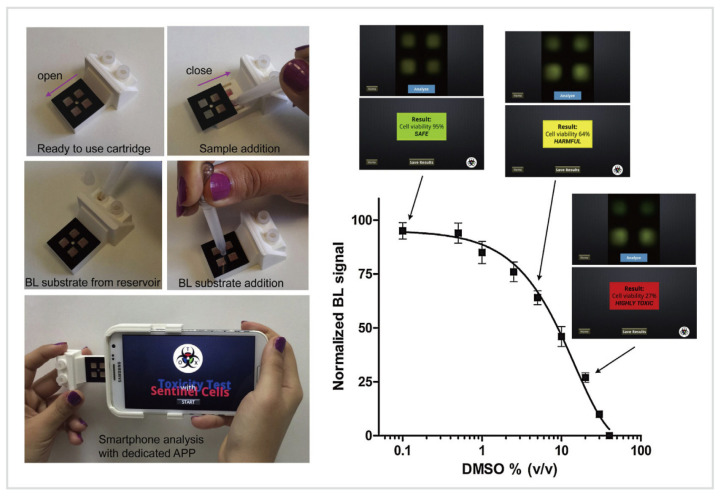
A smartphone-based bioluminescence (BL) whole-cell toxicity biosensor for on-field detection of toxicants; several easy steps required to carry out the toxicity test; dimethyl sulfoxide (DMSO) toxicity curve and the warming message (safe, harmful, or highly toxic) obtained with the smartphone-based biosensor and the Android application (Tox-App), set as “Safe” (cell viability: 100–80%), “Harmful” (79–30%), and “Highly toxic” (<30%) [138]. Copyright 2016, Elsevier.

To facilitate on-field detection of toxicants, Cevenini et al. [138] developed a smartphone-based BL whole-cell toxicity biosensor for real-time, cost-effective, quantitative toxicity testing without trained personnel and laboratory instrumentation (Figure 6). This BL sensing platform consisted of two main components, including a 3D printed BL-cell-contained cartridge for quantification of the BL signals and an Android application, Tox-App, to capture, storage, and process the image, as well as provide real-time toxicity testing results. The human embryonic kidney cells (Hek293T) served as biosentinel cells that expressed a green-emitting luciferase. Dimethyl sulfoxide (DMSO) was used as a model toxic compound, and the percentage of BL signal normalized with respect to control (set as 100%) represented the cell viability value, which delimited the severity of toxicants: “Safe” (cell viability: 100–80%), “Harmful” (79–30%), and “Highly toxic” (<30%). This device provided real-time quantitative results about the toxicity of tested samples, and used BL cells as living biosentinels, enabling battery-free biosensing of toxicants and revealing the great potential of BL cell/bacteria sensor for portable quantitative biosensing.

#### 4.2.3. Colorimetry

Colorimetric sensing modality using soft microfluidics is another way for non-invasive, in situ analysis of biofluids. The colorimetric sensing approach allows inexpensive, rapid, semiquantitative assessment of many sweat biomarkers, such as glucose, urea, lactate, and chloride [21,34]. The biochemical reactions between sweat biomarkers and reagents embedded in sensing platforms yield measurable changes in optical wavelength. Subsequently, capturing the optical information and converting it into quantitative data, such as pH and concentration of the analytes, were accomplished by high-quality digital image capture (e.g., digital camera, smartphone camera) and color extraction (RGB values). Finally, comparing the RGB values extracted from digital images with standard calibration curves measured in the laboratory beforehand can easily determine the concentration of the analytes. As the simplest optical sensing modalities, colorimetric sensing techniques offer multiple attractive features, such as ease of mass-manufacture, simultaneous monitoring of multiple analytes, and ultrathin, lightweight, flexible constructions [73,91,139]. These attributes make them extremely suitable for healthcare monitoring at home or sports analytics in the field. To further illustrate the capabilities of colorimetry, several representative examples will be hereby introduced. 

**Figure 7:**
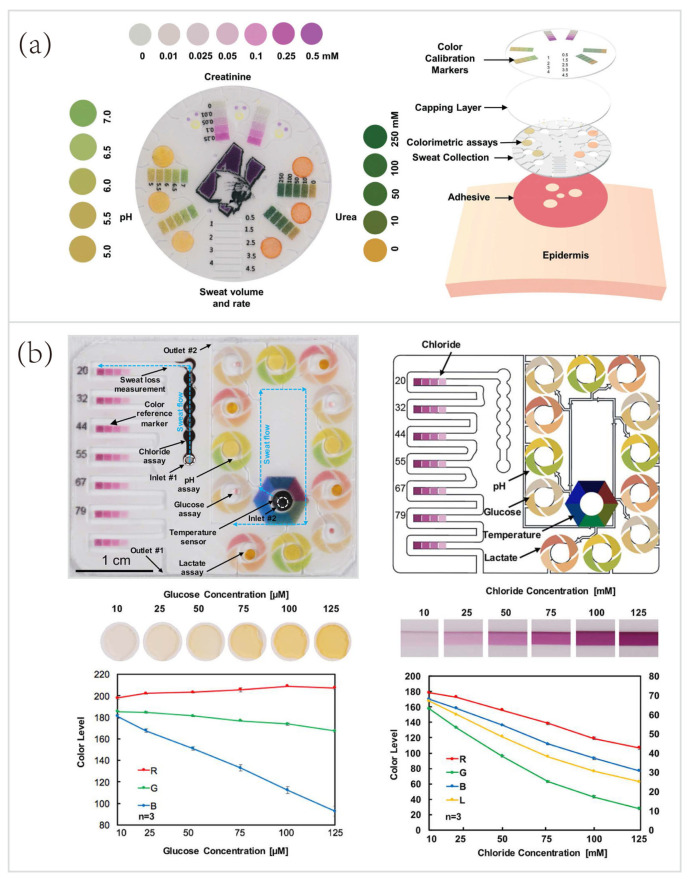
Microfluidic biosensor for colorimetric sensing biomarkers in sweat. (**a**) A soft microfluidic biosensor for colorimetric analysis of biomarkers relevant to kidney function. A top view of its layout and targeted biomarkers, and an exploded view of its different layers and components [34]. Copyright 2019, Royal Society of Chemistry. (**b**) A soft, skin-interfaced multifunctional microfluidic biosensor for accurate colorimetric sensing of sweat temperature, pH, chloride, glucose, and lactate. Two representative optical images of color development of assay microreservoirs as a function of sample concentrations and the corresponding RGB values of glucose and chloride [74]. Copyright 2019, American Chemical Society.

The pioneering work was demonstrated by Koh et al. [21] in 2016, a wearable microfluidic device for colorimetric sensing of sweat. This device integrated four microchannels for guiding the sweat to fill four microreservoirs containing colorimetric reagents for analysis of biomarkers, including glucose, lactate, chloride, and pH. However, this microfluidic biosensor has limitations in accuracy. Recently, considerable efforts have been made to improve the performance of colorimetric sensors based on microfluidics and enzymatic reactions. Zhang et al. [34] established a microfluidic biosensor for colorimetric analysis of biomarkers relevant to kidney function. In Figure 7a, three separate sets of microreservoirs were introduced to achieve the time-sequential analysis of pH and concentrations of urea and creatinine. The printed color reference markers embedded in the capping layer facilitated the quantitative analysis of optical information gleaned by color extraction. This microfluidic biosensor not only improved the accuracy by providing time-sequential results of sweat biochemistry, but also circumvented the requirements for physical exertion by collecting sweat immediately after or during warm-water showering. Hence, this water-proof microfluidic device has vast potential for healthcare monitoring of vulnerable populations, such as infants and the elderly, as well as for sports analytics in aquatic settings [33]. 

Another representative example of colorimetric sensing was a skin-interfaced multifunctional microfluidic system developed by Choi et al. [74] (Figure 7b). This device made good use of similar colorimetric modalities to monitoring pH, chloride, glucose, and lactate. But, importantly, this work further improved the accuracy of colorimetric sensing via its special layout. The color calibration markings laminated onto the top surface of the system, to surround each of the microreservoirs or each segment of the serpentine microchannel, as shown in Figure 7b. This layout facilitated the real-time quantitative analysis in various lighting conditions (such as under natural ambient conditions), which increased the accuracy of optical information gleaned from digital image analysis. The bottom two images showed in Figure 7b were the optical images of the color development of microreservoirs as a function of sample concentrations and the corresponding RGB values of glucose and chloride. The spectral information for color reference markers was obtained from a series of in vitro tests with standard samples of simulated sweat. Moreover, this platform also exploited thermochromic materials, a ternary cholesteric liquid crystalline mixture, for real-time temperature sensing (ranging from 31 to 37 °C) of sweat as it generated from the skin. Compared to electrochemical sensing modality, colorimetric sensing provides an easier way to monitoring sweat biochemistry and thermal conditions without the need for wearable power source, in situ data processing, and wired/wireless data transmission. 

#### 4.2.4. Optoelectronic Sensing

**Figure 8:**
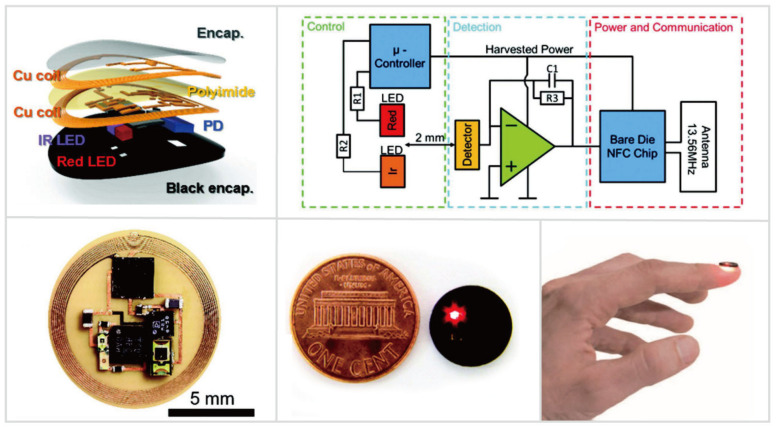
A novel optoelectronic wearable biosensor for wireless, battery-free pulse oximetry. A schematic illustration of the layout of the fingernail-mounted pulse oximetry, and a block diagram of the functional components; images of an unencapsulated device, a device next to a US one-cent coin, and a fingernail-mounted device during operation [26]. Copyright 2017, Wiley.

Advanced body-worn optoelectronic devices have been designed for quantitative, continuous sensing of vital signs. Recently, Kim et al. [26] reported an optoelectronic biosensor for pulse oximetry (Figure 8). Due to the high-power consumption of the LEDs, conventional pulse oximeters either rely on the bulky battery or hard-wired power supply [52]. To realize the on-field tests, wireless, battery-free, fingernail-mounted pulse oximetry was developed. This miniaturized oximeter exploited the bilayer loop antenna configured to maximize the efficiency of power harvesting and the distance for wireless data transmission. Wireless delivered power was used to drive a microcontroller and an operational amplifier. The infrared and red LEDs emitted light chronologically under the control of the microcontroller. The intermediate photodetector captured the backscattered light and an analog-digital converter amplified and digitized the resulting signals. Ultimately, the data of blood oxygenation (SpO_2_), heart rate (HR), and heart rate variation (HRV) was wirelessly transmitted via NFC chipsets. This fingernail-mounted oximeter extends the mounting time up to 3 months and minimized the risk for irritation and discomfort. Long-term non-irritating integration with the human body allows for continuous monitoring of athletes’ cardiovascular physiology, enabling easier management of daily athletic training and progress. Besides, “fingernail-mounted” modalities show several unnoticed but outstanding merits over “skin-interfaced” modalities. Compared to the skin, the fingernail can be exploited as a mounting location for long-term biosensing due to its optical transparency, mechanical rigidity, minimal interfacial water loss, and absence of nerve endings.

### 4.3. Other Sensing Modalities

Although the notable advances reported in the above-mentioned studies demonstrate the excellent performance of skin-interfaced wearable sensors for sweat biosensing, other sensing modalities still hold great potential. Over the past decade, novel sensing modalities emerge quickly, revealing promising solutions for long-term, non-irritating, and personalized therapeutics. Various mounting locations (e.g., tooth, fingernail) [26,52,53], integration methods (e.g., wearable, implantable) [140,141,142,143], and sensing modalities (e.g., volumetric, ultrasonic and optofluidic) [53,140,144] have opened up a spectrum of applications in wearable/ambulatory healthcare and sports analytics. Novel biosensing modalities for promoting these fields are urgently required, but herein we only present one novel sensing modality, volumetric sensing, to show the enormous potential of wearable biosensing. 

#### Volumetry

Microfluidic systems demonstrate great potential for wearable biosensing. In the past five years, an innovative biosensing modality—volumetric biosensing, a quantitative method to ascertain sweat loss/rate for reflecting users’ hydration status and providing suited rehydration strategies—quickly emerged. Herein, volumetric sensing of sweat will be introduced with two illustrative examples. Soft microfluidic biosensors make use of the pressure naturally-induced by the sweat glands to drive sweat through a series of microchannels to fill sweat collecting channels time-sequentially for real-time quantification of sweat loss/rate [63,145]. The microfluidic platforms, for volumetric sensing of sweat over local anatomical regions, usually consist of opening(s) for defining the sweat collecting area(s) where sweat can enter the platform, water-soluble dye(s) inserted adjacent to the inlet(s) to facilitate real-time visualization of sweat loss, and the serpentine microchannel(s) for sweat volumetry [21,34,43,74]. Finally, volumetric sensing of the colored sweat collected by the microchannel(s) is performed by digital image capture and analysis. Wearable biosensors for on-field tests usually exploit smartphone cameras for quantification of sweat loss/rate by image processing and real-time display of the data for end-users [21,27,73]. 

**Figure 9:**
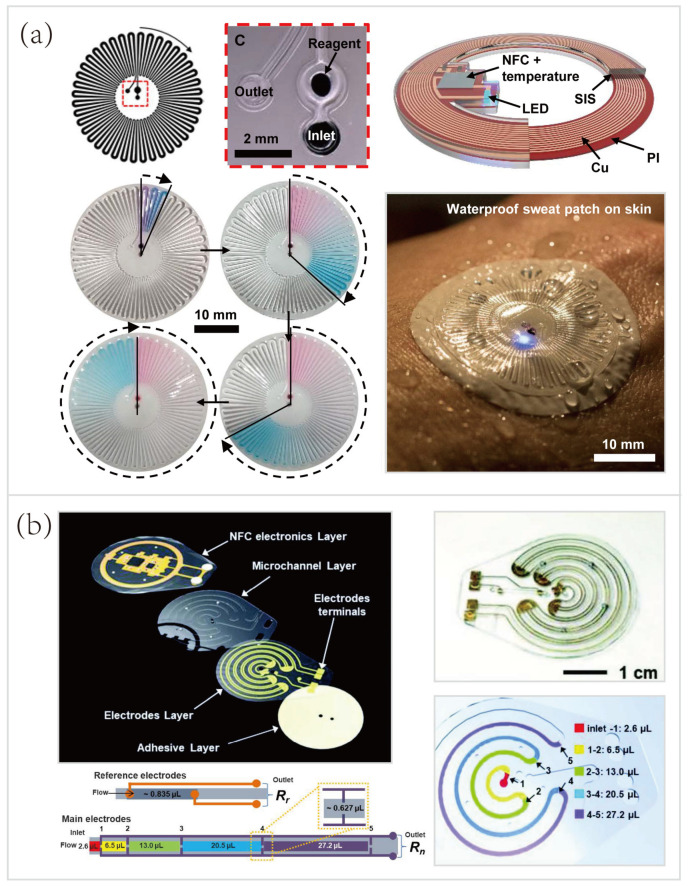
Wearable biosensors for sweat volumetry. (**a**) A waterproof skin-interfaced microfluidic biosensor for volumetric sensing, biomarker analysis, and thermography in aquatic settings; images showing the special inlet and outlet pores, as well as the volumetric sensing by using red/blue dyes to facilitate the visual inspection [33]. Copyright 2019, American Association for Advancement of Science. (**b**) A microfluidic/electronic biosensor for digital tracking of sweat loss/rate and electrolyte composition; illustrations showing the volumes of microfluidic channels that lay between different pairs of probing pads [27]. Copyright 2018, Wiley.

In recent years, various microfluidic platforms, characterized by different microchannel layouts and geometries, have been developed for volumetric sensing of sweat [43]. But, few of them can operate in aquatic settings (such as during showering, swimming, or diving). To fulfill the requirements of sweat volumetry for athletes participating in aquatic sports, Reeder et al. [33] developed a waterproof skin-interfaced microfluidic biosensor for sweat volumetry, biomarker analysis, and thermography (Figure 9a). Underwater sweat collection and volumetric sensing of sweat loss/rate were introduced. The special microfluidic inlet and outlet pores were essential for sweat loss visualization and device’s waterproofing. As shown in Figure 9a, the water-soluble reagents, consisting of a food dye to facilitate the visual inspection of sweat collection, resided in the microchamber adjacent to the inlet pore. This food dye contained red/blue particles with different dissolution rates that generated a volume-dependent color gradient as the microchannel filled with sweat. The serpentine microchannel has 40 turns, each of which has a volume of 1.5 μL. Thus, the sweat loss/rate can be quantified by counting the sweat-filled turns. Besides, this device contained only one tiny outlet and a small amount of “dead volume” near the outlet pore, eliminating the interference from the outside aquatic environment, which was considered a highly attractive feature for aquatic sports analytics.

Another research presented an alternative method, instead of using colored dyes, to quantify sweat loss/ rate. Kim et al. [27] established a microfluidic/electronic biosensor for digital tracking of sweat loss/rate and electrolyte composition. As shown in Figure 9b, this platform also deployed a serpentine microchannel for volumetry of collected sweat, but a single pair of electrodes with five pairs of probing pads were used to trace the collected sweat. The total resistance (*Rn*) across the main electrodes depended on the number of pairs of probing pads that were bridged by sweat. The bottom two schematics of Figure 9b showed the correlations between the number of pairs of probing pads bridged by collected sweat and the amount of sweat collected by the microfluidic channel. This novel approach circumvents the need for digital image capture and analysis, which seemingly simplifies sweat volumetry. However, the volumes of microchannels between pairs are significantly different (from 2.6 μL to 27.2 μL). Despite the flaw mentioned here, future research efforts on the device design may provide a possible solution to equal the volumes of microchannels between different pairs of probing pads. Hence, optimizations of the geometry of the microfluidic channel and the corresponding layout of the electrodes present a promising direction for the improvement of digital sweat volumetry. 

## 5. Key Analytes and Wearable Biosensing Platforms

### 5.1. Key Analytes in Wearable Biosensing

Biofluids are largely unexplored resources that contain a rich milieu of important biomarkers, from electrolytes [14,61,126], metabolites [21,34], heavy metals [31], cytokines [146,147], hormones [80,148,149] and amino acids [150] to exogenous drugs [77,83], each of which provides valuable insights into physiological status, health conditions, pharmacokinetics, and sports performance. Sweat and ISF are two representative biofluids that offer great potential for home-based healthcare monitoring or on-field sports performance assessment due to ease of non/minimally-invasive collection and analysis by using wearable biosensors [15,41,81]. Although traditional wearable platforms have demonstrated the effectiveness of measuring a few biophysical markers [25,49,50], skin-interfaced biosensors mainly exploit valuable resources—sweat and ISF biochemical markers for healthcare and sports monitoring as shown in Table 1. But, due to the space limitation, only the key sweat targets will be discussed herein, the key ISF analytes and a few wearable biosensors for ISF analysis will be presented in a limited manner in Section 5.3. 

Electrolytes, the most abundant analytes in sweat, are widely tested in wearable biosensing [151]. Significant interest in Na^+^, K^+^, NH_4_^+^, and Ca^2+^ stems from their applications in disease diagnostics and sports analytics. For example, the breakdown of proteins can result in the presence of ammonium in blood before the liver converts them to urea [68]. There, high levels of NH_4_^+^ in excreted sweat can be used as indicators of hepatic diseases, such as hepatitis, cirrhosis, and related disease—hepatic encephalopathy (HE) [35,152]. Kim et al. [35] reported a wearable microfluidic biosensor with integrated enzymatic reactions for colorimetric sensing of the concentration of ammonia and ethanol in sweat. This sweat biosensor could serve as a diagnostic tool of HE. Besides, ionized calcium is another important marker of homeostasis. Excessive variations of Ca^2+^ levels in biofluids are detrimental to the human body, causing myeloma, renal failure, kidney stones, and hyperparathyroidism [32]. Moreover, electrolytes, especially Na^+^ and K^+^, play a critical role in maintaining the electrolyte balance and hydration status. Excessive loss of them could result in hyponatremia, hypokalemia, and muscle cramps, leading to inferior sports performance [14]. 

Metabolites are rich solutes in sweat which are highly attractive for diverse applications in wearable disease diagnostics and sports analytics. For instance, glucose and lactate attract most research efforts [45]. Real-time tracking of glucose concentration in biofluids not only has utility in the diagnosis of diabetes mellitus [153], but also can reflect energy availability and consumption for athletic monitoring [43]. Likewise, sweat lactate represents a key indicator of muscle fatigue, tissue hypoxia, and exercise intensity [21,75]. Thus, many wearable biosensors capable of simultaneously monitoring sweat glucose and lactate have been successfully developed [14,21,73,74,75], because of the importance of continuously gleaning metabolic information from sweat. Additionally, another three sweat metabolites, including urea, uric acid, and creatinine, create opportunities for diagnostics of kidney disorders. The levels of sweat urea and creatinine are typically higher in patients with kidney disorders than in healthy individuals, since the failure of glomerular function [79,154]. As described previously, Zhang et al. [34] reported a microfluidic biosensor for colorimetric analysis of urea and creatinine for POCT of kidney diseases. Similarly, newborns usually undergo sweat chloride tests for the diagnosis of cystic fibrosis (CF) [69,155].

Other key targets, such as hormones [80,148,149], cytokines [146], heavy metals [31,61,76], and exogenous drugs [83,156], have also attracted considerable attention in wearable biosensing for healthcare monitoring. As such, cortisol is a steroid hormone in sweat, and recent efforts have been made in wearable cortisol monitoring for stress level tracking and disease diagnostics, including Cushing’s syndrome and Addison’s disease [80,148,149]. Besides, cytokines and heavy metals have also been non-invasively analyzed by wearable sensors to gain insights into inflammatory response [146,147], heavy metal exposure and detoxication [31], respectively. Moreover, wearable biosensors have been exploited to monitoring exogenous drugs, such as Levodopa and caffeine [77,156]. Wearable biosensing has a considerable potential to transform the current state-of-the-art precision medicine and drug delivery research [83]. The concentration of drug in biofluids (especially sweat and ISF) gleaned from wearable monitoring can serve as feedback to improve personalized drug dosage, to establish closed-loop therapeutics, and to provide raw data for pharmacokinetics [77,153,157]. 

### 5.2. Wearable Sweat Biosensing Platforms for Healthcare and Sports Monitoring

The rich composition of sweat analytes makes the wearable sweat biosensing platform applicable to healthcare and sports monitoring. Over the past decade, an increasingly large number of wearable biosensors have been developed, as we systematically reviewed in Section 4. However, the potential impact of wearable sweat biosensing technologies on sports analytics has not yet been fully recognized. To broaden the scope of wearable sweat sensing (like other analytes, integrated biosensors), several wearable platforms will be briefly introduced herein. 

**Figure 10:**
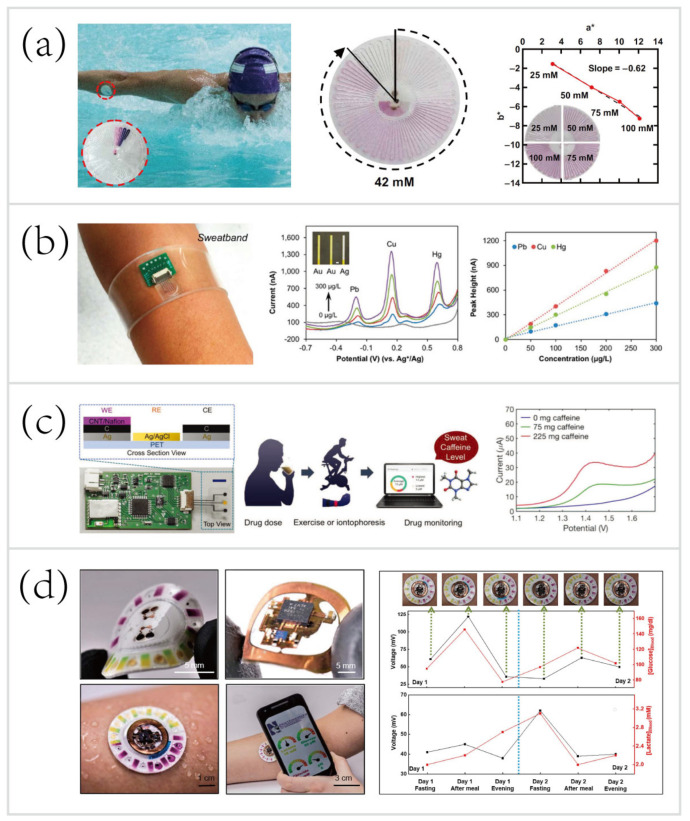
Wearable sweat biosensing platforms for healthcare and sports monitoring. (**a**) A waterproof skin-interfaced microfluidic biosensor for sweat chloride analysis in aquatic settings [33]. Copyright 2019, American Association for Advancement of Science. (**b**) A wearable biosensor for multiplexed monitoring of heavy metals, including Zn, Cd, Pb, Cu, and Hg [31].Copyright 2016, American Chemical Society (**c**) A wearable biosensor for methylxanthine drug (caffeine) monitoring [156]. Copyright 2018, Wiley. (**d**) A skin-mounted hybrid sensor for analysis of sweat glucose, lactate, pH, and chloride; correlations of data acquired from sweat biosensors (black line) with that acquired from blood glucose and lactate meters (red line), respectively [73].Copyright 2019, American Association for the Advancement of Science.

In one example, Reeder et al. [33] developed a waterproof microfluidic platform for sweat volumetry, biomarker analysis, and thermography in aquatic settings (Figure 10a). For biomarker analysis, colorimetric sensing was introduced: the reagent resided in the adjacent chamber reacted with sweat to create a colorimetric response corresponding to the concentration of chloride. A printed color reference dial at the center facilitated visual inspection. Besides, two representative biosensors exploited electrochemical sensing for in situ monitoring of heavy metals—Zn, Cd, Pb, Cu, and Hg [31] (Figure 10b), and an exogenous drug—caffeine [156] (Figure 10c). These systems based on electrochemical biosensing are characterized by high selectivity, sensitivity, and reproducibility. Tai et al. [156] reported a wearable biosensor with the capability of methylxanthine drug monitoring by electrochemical DPV, which is an important step to complement the traditional method of drug dosage tracking—blood sampling and analysis. Sports medicine personnel could be specifically interested in this work due to their ability to monitor the drug dosage during exercise intervention of the diseased. Another example of using wearable biosensors for drug monitoring was combined with microneedles technologies, and will be described in the following Section 5.3.1.

Recent progress has led to the advent of hybrid biosensors, which can monitor multiple parameters by simultaneously performing electrochemical, colorimetric, and volumetric sensing. To yield a comprehensive evaluation of the physiology and sports performance of end-users, Bandodkar et al. [73] developed a skin-mounted microfluidic/electronic biosensor for simultaneous monitoring of chloride, pH, lactate, glucose, and sweat loss (Figure 10d). The hybrid biosensor contained a reusable, thin NFC subsystem and a soft, disposable microfluidic subsystem. The NFC subsystem allowed for electrochemical sensing in a mode that targeted glucose and lactate, and spontaneously generated electrical signals proportional to their concentrations. The microfluidic subsystem embedded colorimetric reagents for tracking the pH and concentration of chloride, with an additional ratcheted microchannel for quantification of sweat rate and total sweat loss. As shown in Figure 10d (right), a field study aimed to suggest the potential for acquiring semi-quantitative data by analyzing sweat biochemistry. This study focused on comparing temporal variations in glucose and lactate levels in blood and sweat due to exercise engagement or food intake. The results showed a comparison between the data acquired from hybrid biosensors with the data acquired from blood glucose and lactate meters, respectively. Over two days, the blood glucose and lactate levels after each session followed trends that qualitatively similar to those of data measured in sweat. These correlations were confirmed by literature [69,153,176,177,178]. 

### 5.3. Microneedles Platforms

Several decades ago, microneedles (MNs) were first introduced to perform transdermal drug delivery [179]. MNs with small length (usually less than 1000 μm [180,181]) penetrate the stratum corneum and open windows for the ISF biosensing or form transient microchannels for drug delivery while preventing the nerves and blood vessels from stimulation and impairment. Currently, MNs have been utilized for applications in the following areas: ISF sampling [180,182], disease diagnostics [153,183,184], drug delivery [179,185,186], cosmetics [181,187,188,189], etc.

MNs have wide applications and show tremendous promise for applying biomedical advances into wearable healthcare and sports monitoring due to the three following intrinsic advantages.

1. Enhanced compliance. The patients and athletes will not suffer from pain, discomfort, and needle-phobia [179,180,190,191].
2. Easy-to-use. MNs are user-friendly biosensing and drug delivery devices that can be directly applied to the skin. Conventional methods, hypodermic injections, conversely, demand professional personnel who undergo rigorous medical training [179].
3. Real-time in situ biosensing and controllable/long-term drug delivery. MNs realize pain-free, in situ diagnostics even combine with feedback-based long-term drug delivery [160].

Recent advances in wearable MN patches capable of minimally-invasive, transdermal sensing biofluids [153,160,179,192,193] and pain-free drug delivery [184,194,195] provide an opportunity to perform sports monitoring, especially beneficial to sports nutrition and sports medicine [196]. These techniques combined with electrochemical/microfluidic biosensing modalities are expected to lead to several breakthroughs in wearable biosensing. 

#### 5.3.1. Microneedles for Transdermal Biosensing

In recent years, although the majority of MNs are still focused on painless drug delivery, MNs for transdermal biosensing are promising and receive considerable attention [190]. ISF contains rich physiological/pathological information than ever envisaged [197], in the meantime, the MN-based biosensor can serve as a portal for the pain-free acquisition of valuable information. Besides, recent research efforts have led to the emergence of advanced microfabrication and biophotonics that facilitate the manufacture of MNs [37,39,198]. More complicated MN platforms that integrate electrochemical biosensors [157,169,181] or biofuel cells [37] (harvesting energy from biofluids, such as sweat and ISF, to power wearable platforms) have been manufactured. These integrated biosensors demonstrate great promise for monitoring physiological status and athletic performance in field tests, as well as detecting doping in sports competitions.

**Figure 11:**
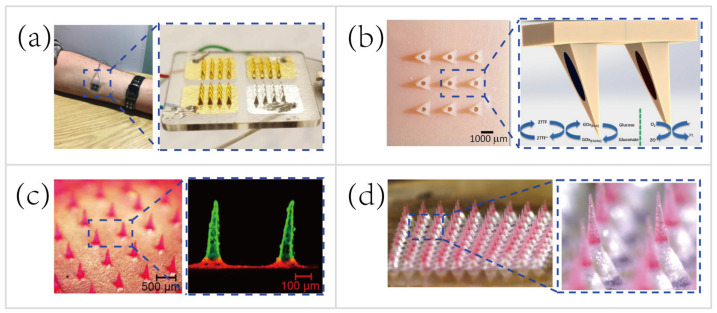
Representative applications of microneedles (MNs) for transdermal biosensing and pain-free drug delivery. (**a**) MN-based biosensor for continuous monitoring of β-lactam antibiotic concentration in vivo [83]. Copyright 2019, American Chemical Society. (**b**) MN-based self-powered glucose sensor and schematic reaction on two of the hollow MNs located within the MNs array [37]. Copyright 2014, Elsevier. (**c**) Multilayered pyramidal dissolving MNs, composed of silk fibroin tips supported on flexible pedestals, for improving drug delivery [38]. Copyright 2017, Elsevier. (**d**) MN patch with drug-loaded tips and effervescent backings for long-acting reversible contraception [40]. Copyright 2019, American Association for the Advancement of Science.

MNs only penetrate the stratum corneum of the skin, therefore do not reach the capillary to sample blood for biosensing. Instead of hypodermic needles that sample blood for medical testing, the transdermal MN-based biosensors adopt a minimally-invasive way to sample the ISF for therapeutic drug monitoring (TDM). Using the TDM to optimize the drug dosage is important for improving therapeutic efficacy and predicting any adverse outcome, such as resistance and toxicity [199]. For example, Gowers et al. [83] developed a TDM system (Figure 11a), an MN-based biosensor for real-time continuous monitoring levels of β-lactam antibiotics in vivo. The MNs were coated with multiple layers, including a gold working electrode layer, a pH-sensitive iridium oxide layer, a β-lactamase hydrogel layer, and a poly(ethylenimine) layer. The pH-sensitive layer detected changes in local pH as a result of β-lactam hydrolysis with the presence of β-lactamase. Afterwards, the potentiometric modality was applied for establishing the relationship between variations in local pH and the potential measured. Such a biosensor is capable of real-time tracking penicillin concentrations, paving the way for personalized drug dosage that is a large step towards precision medicine. But, these MN-based biosensors are still at an early stage of development. A daunting challenge still exists, namely the need for a continuous, reliable power supply. 

A majority of wearable biosensing devices rely on the external power supply [14,26,88,90,153], which causes numerous technical difficulties in the field tests. Thus, researchers begin to seek new methods to obviate the need for an external power supply. Bandodkar et al. [200] made a major thrust of biofuel-cell (BFC) wearable devices that scavenged energy from human sweat. The wearable energy harvester they developed converted readily available sweat lactate into electricity. Lightweight, ultrathin, stretchable, high power density were the attractive features of this wearable harvester. Nowadays most MN-based sensing patches are still relying on the external power supply [83,153,201]. To overcome this major drawback, Valdés-Ramírez et al. [37] developed an MN-based self-powered BFC glucose biosensor that harvested biochemical energy from the wearer’s ISF (Figure 11b). The self-powered biosensor harnessed glucose as the fuel to eliminate the need for an external power supply, and also provided power signals proportional to the glucose concentration. Besides, this self-powered MN-based sensor displayed high selectivity and stability that showed considerable promise for battery-free transdermal glucose monitoring. In short, the combination of energy harvester, electrochemical sensor, and MN technology will address some key challenges, such as integrating monitoring multiple analytes in ISF and automatic feedback-based transdermal drug delivery using MN-based self-powered BFC biosensors.

#### 5.3.2. Microneedles for Pain-free Drug Delivery 

Microneedles for transdermal drug delivery reduce the systemic side effects and improve dosage efficacy and patient compliance [180] compared to hypodermic needles [38,180,202]. Typically, external small molecules (e.g., drug contents) can be minimally-invasive delivered percutaneously harnessing passive mass transfer. To facilitate the establishment of closed-loop diagnostic and therapeutic systems, MNs for drug delivery are also attractive, especially for controlled or long-term non-irritating drug release. In the past few years, great efforts have been made to find a way for controllable/long-term drug delivery using MNs, such as thermo-responsive MNs [153], stretch-triggered MNs [196], multilayered MNs [38], and effervescent MNs [40]. 

To deliver the drug contents in a controllable manner, Lau et al. [38] developed a multilayered pyramidal dissolving MN patch with flexible pedestals (Figure 11c). The drug release time and rate were regulated by the dissolution rate of diverse biomaterials in different layers of the MNs. Hence, the MNs were applicable to control the release rate of the anti-inflammatory drug to facilitate personalized sports medicine. For instance, the rapid dissolution of the tip’s outermost layer of the multilayered pyramidal MNs can quickly control inflammation and continuously treat chronic inflammation via sustained dissolution of the inner layer. 

Another group demonstrated the pursuit of long-term personalized drug delivery. Wei Li et al. [40] developed an effervescent system to increase access to long-acting contraception (Figure 11d). The MNs consisted of drug-loaded tips and effervescent backings containing polyvinylpyrrolidone (PVP), sodium bicarbonate, and citric acid. Once inserted in the skin, the ISF solubilized the effervescent backing and the CO_2_ generated from the reaction of citric acid with sodium bicarbonate facilitated the separation of the interfaces between the MNs’ tips and patch backings (detachment efficiency of 91.7 ± 2.4%). Long-acting contraception was achieved in vivo by the dissolution of MNs’ tips to release levonorgestrel (LNG) for 2 months and maintained the LNG concentrations above the human contraceptive threshold level for >1 month (after rats being administered MNs). 

In sum, MNs are not only appealing avenues to interrogate the ISF for biosensing, to carry out controllable/long-term transdermal drug delivery [179,185,186], but also can facilely incorporate with electrochemical biosensors [157,169,181] to realize closed-loop personalized diagnostics and therapeutics [153,193,203]. Moreover, recent advances in biofuel cells [89,200,204,205,206] could further address the key issue, lack of thin and reliable wearable power sources. These eminent features would enable a broad range of important applications in the sports field where real-time athletic performance monitoring and personalized sports injury treatments are urgently demanded [196]. We envisage that the advancement of MNs for biosensing and drug delivery will encourage the emergence of new generations of wearable athletic biosensors, that would revolutionize sports monitoring and sports medicine in the near future. 

## 6. Unsolved Challenges for Future Research

Although considerable research efforts and remarkable progress have been made in wearable biosensing for healthcare monitoring and sports analytics, especially the field of microfluidics for sweat biosensing, several technical difficulties remain in aspects related to lack of well-established gold standard, as well as the selectivity, sensitivity, power supply, integration, cost of the device. First of all, unlike blood tests that have numerous well-established clinical gold standards, such as diabetes diagnosis (glucose concentration ≥ 7 mmol/L) [207], it is particularly difficult to establish gold standards for wearable biosensing of non-blood biofluids. Take sweat biosensing, for example. The sweat loss and the concentration of biomarkers show wide variations between different regions of the body. Besides, acquiring reliable concentrations of biomarkers (especially for scarce analytes) is challenging due to the relatively small amounts of collected sweat compared to total sweat loss. The calibration of biomarker concentrations is equally challenging since their strong relation to individual hydration status. Therefore, additional advances in wearable biosensors are needed, especially those related to diagnostic applications. 

Furthermore, most wearable devices, especially electrochemical biosensors, rely on the continuous, reliable power supply, including miniaturized batteries, wireless power delivery devices, and biofuel-cell (BFC) energy harvesters. Miniaturized batteries, both coin cell and flexible batteries, have poor performance, requiring frequent recharging or large power source carried by wearers. The alternative, wireless power harvested from NFC chipsets, also has a tough problem that proximity (<1 m) is required for power delivery. Biofuel-cell energy harvesters scavenging energy from sweat or ISF metabolites (e.g., lactate, glucose) have some potential but power density still needs to elevate. Additionally, wearable biosensors, such as soft, skin-interfaced microfluidic/electrochemical biosensors, have to in situ monitor multiple analytes at extremely low concentrations without sample pretreatment. Thus, sensitivity and selectivity of biosensors are considered fundamental requirements for wearable healthcare monitoring and sports analytics. Besides, in situ monitoring biomarkers and real-time data acquisition demand the integration of monitoring subsystem, data processing subsystem, and wireless data transmission subsystem. This integration will yield significant additional expenditures and difficulties. More importantly, a majority of wearable biosensors meet difficulties to re-use since they do not discern the disposable and re-useable components of wearable biosensors. Nonetheless, future studies may focus efforts to design cheap, single-use plug-in parts of biosensors, which will explore possible avenues for the investigation and commercialization of this fascinating technology.

Unmet challenges also exist in the translation of biomedical advances into sports analytics. As mentioned previously, a vast majority of wearable technologies focus on healthcare monitoring. Firstly, well-established biomedical techniques have not yet been broadly applied in the sports field, since the enormous potential of the wearable biosensor for sports analytics has not been recognized by most researchers (e.g., biomedical engineers). Unfortunately, innumerable multidisciplinary studies concerning wearable technologies have been conducted within the medical and engineering fields instead of outside fields, such as sports analytics or sports engineering. Secondly, current existing sports analytical techniques, especially for chemical analysis of biofluids, are confined to the laboratory or hospital settings. Specifically for sports biochemists, difficulties largely follow from lack of engineering background and expertise, few opportunities for interdisciplinary collaborations. Ultimately, although several research groups at Northwestern University, University of California (Berkley) gain opportunities for in-depth interdisciplinary collaborations and develop numerous wearable biosensors for sports analytics, an obstacle, lack of practical data interpretation, in the path of the widespread application and commercialization of wearable biosensors still stubbornly persists. The wearable biosensors they developed have increasingly high performance related to sensitivity, selectivity, accuracy, and multifunctionality. Nevertheless, even if accurate data of sweat biomarkers gleaned by wearable biosensors can be facilely collected, the concentrations of sweat metabolites and electrolytes can be easily displayed on the smartphone, the end-users still do not know what these mean and how these data represent their health status and sports performance. Therefore, what next for wearable biosensors, from healthcare monitoring to sports analytics? It is perhaps a revolutionary technology for the future, although a great deal of work needs to be done before it achieves prosperity in academia and success in commercialization. 

## 7. Conclusions and Perspectives

Recent advances in stretchable materials, microfluidics, optical/electrochemical sensors, image processing and analysis, NFC and wireless power supply, microneedles, as well as big data and cloud computing provide a robust foundation for wearable biosensing, especially the field of microfluidics for sweat and ISF biosensing. The emerging sampling and sensing modalities for obtaining sweat biochemical information offer profound insights into human physiology, metabolism, and sports performance, which complement traditional biophysical sensing modalities. Representative examples of wearable biosensors capable of in situ monitoring sweat biomarkers, from electrolytes, metabolites, heavy metals, cytokines [146], hormones [80,148,149], amino acids [150] to exogenous drugs [83,156], provide a window on the valuable biochemical resources that have a substantial impact on disease diagnostics, precision medicine, drug delivery, and sports analytics. However, the majority of advanced wearable sweat biosensors still focus on healthcare monitoring to improve home-based disease diagnosis. Although wearable biosensors for sports analytics are underdeveloped, the transition from focusing on healthcare monitoring to combining with sports analytics is an important step in a trend towards better preventive healthcare and sports & health science. It offers revolutionary capacities for future wearable technologies. It also in its infancy, and a great deal of work still needs to be done, such as integration of well-developed technologies (e.g., ultrasonics, implants, optofluidics, optoelectronics, microneedles, etc.), for building up a picture of future biosensing technologies. Despite the remarkable progress that several research groups achieved over the past five years, daunting challenges remain in the power supply, data interpretation, the cost of mass-fabrication, as well as in-depth interdisciplinary collaboration. With these challenges overcome, the commercialization and widespread adoption of wearable biosensors in sport-related fields will be fully realized, and state-of-the-art athletic monitoring and sports analytics will be transformed fundamentally.

## Figures and Tables

**Table 1:** Key sweat and ISF analytes in wearable healthcare and sports monitoring.

Key Targets	Analytes	References	Representative Applications
Electrolytes	^1^ Na^+^	[14,61,126]	Disease diagnostics (Ca^2+^ for kidney stones and hyperparathyroidism; NH_4_^+^ for ^3^ HE) Sports analytics (Na^+^ and K^+^ for electrolyte balance, muscle cramps, and de/rehydration)
	^1,2^ K^+^	[158,159,160]	
	^1^ NH_4_^+^	[35,68,161]	
	^1^ Ca^2+^	[32,161,162]	
Metabolites	^1,2^ Glucose	[37,75,153]	Disease diagnostics (glucose for diabetes, chloride for cystic fibrosis; urea, uric acid, and creatinine for kidney diseases, such as uremia; cholesterol for atherosclerosis) Sports analytics (lactate for muscle fatigue, tissue hypoxia, and exercise intensity; glucose for energy availability and consumption)
	^1,2^ Lactate	[74,127,163]	
	^1^ Creatinine	[21,34]	
	^1^ Chloride	[33,69,74,91]	
	^1^ Uric acid	[150,164,165]	
	^1^ Urea	[34,166]	
	^2^ Cholesterol	[167]	
Heavy metals	^1^ Zn	[31,61,76]	Monitoring of heavy metal intake and detoxification (excess or deficiency) Determination of heavy metal exposure
	^1^ Cu,^1^ Cd,^1^ Pb,^1^ Hg	[31]	
Cytokines	^1,3^ IL-1β	[146]	Monitoring of inflammation (CRP, TNF-α) Precision medicine (IL-1β for ^3^ IBD treatment)
	^1,3^ CRP	[146]	
	^1,2,3^ TNF-α	[147,168]	
Hormones	^1^ Cortisol	[80,148,149]	Disease diagnostics (Cushing’s syndrome, Addison’s disease) Mental health management (stress levels)
Amino acids	^1^ Tyrosine	[150]	Disease diagnostics (liver diseases, traumatic brain injury, Alzheimer’s disease) Nutritional management (eating disorders)
	^2^ Glutamate	[169]	
Exogenous drugs	^2^ β-lactam	[83]	Precision medicine (personalized drug dosage, closed-loop therapeutics) Drug delivery (pharmacokinetics, therapeutic drug monitoring for antibiotics)
	^2^ Vancomycin	[170]	
	^1^ Levodopa	[77]	
	^1^ Caffeine	[156]	
Others	^1,2^ Ethanol	[35,171,172]	Alcohol consumption analysis
	^1,2^ pH	[32,34,74,173]	Sports analytics (electrolyte balance) Disease diagnostics (metabolic alkalosis)
	^1^ Sweat loss/rate	[21,33,34,73]	Sports analytics (de/rehydration) Disease diagnostics (autonomic nervous disorders; hyper/hypohidrosis)
	^2^ Immunoglobulin	[174,175]	Early disease detection Investigation of vaccine effectiveness

Analytes sources: ^1^ sweat and ^2^ interstitial fluid (ISF); ^3^ Abbreviation: HE, hepatic encephalopathy; IL-1β, Interleukin-1β; CRR, C-reactive protein; TNF-α, Tumor necrosis factor-α; IBD, Inflammatory bowel disease.
